# A Systematic Review of Reviews of Correctional Mental Health Services Using the STAIR Framework

**DOI:** 10.3389/fpsyt.2021.747202

**Published:** 2022-01-18

**Authors:** Alexander I. F. Simpson, Cory Gerritsen, Margaret Maheandiran, Vito Adamo, Tobias Vogel, Lindsay Fulham, Tamsen Kitt, Andrew Forrester, Roland M. Jones

**Affiliations:** ^1^Forensic Psychiatry, Centre for Addiction and Mental Health, University of Toronto, Toronto, ON, Canada; ^2^Department of Forensic Psychiatry, Centre for Addiction and Mental Health, University of Toronto, Toronto, ON, Canada; ^3^Centre for Addiction and Mental Health, Toronto, ON, Canada; ^4^Department of Psychology, Centre for Addiction and Mental Health, University of Toronto, Toronto, ON, Canada; ^5^Forensic Psychiatry, Department of Psychological Medicine and Clinical Neursciences, School of Medicine, Cardiff University, Cardiff, United Kingdom

**Keywords:** prison, systematic review, mental health care, STAIR model, screening

## Abstract

**Background:**

Rising demand for correctional mental health services (CMHS) in recent decades has been a global phenomenon. Despite increasing research, there are major gaps in understanding the best models for CMHS and how to measure their effectiveness, particularly studies that consider the overall care pathways and effectiveness of service responses. The STAIR (Screening, Triage, Assessment, Intervention, and Re-integration) model is an evidence-based framework that defines and measures CMHS as a clinical pathway with a series of measurable, and linked functions.

**Method:**

We conducted a systematic review of the reviews of CMHS elements employing PRISMA guidelines, organized according to STAIR pillars. We assessed the quality of included studies using the AMSTAR-2 criteria. Narrative reviews were read and results synthesized.

**Results:**

We included 26 review articles of which 12 were systematic, metaanalyses, and 14 narrative reviews. Two systematic reviews and seven narrative reviews addressed screening and triage with strong evidence to support specific screening and triage systems. There was no evidence for standardised assessment approaches. Eight systematic reviews and seven narrative reviews addressed interventions providing some evidence to support specific psychosocial interventions. Three systematic reviews and six narrative reviews addressed reintegration themes finding relatively weak evidence to support reintegration methods, with interventions often being jurisdictionally specific and lacking generalizability.

**Conclusions:**

The STAIR framework is a useful way to organize the extant literature. More research is needed on interventions, assessment systems, care pathway evaluations, and reintegration models.

## Introduction

Rising prison populations internationally have been a source of major concern (1). Although the percentage of prison inmates who have a serious mental illness (SMI) has been relatively static over time at 15% (2), increasing prison musters mean there are many more people with SMI in custody (1, 3). Historically, there has been low access to mental health care in custody and few benchmarks to measure the adequacy of services (4). Human rights standards [for instance UNDOC (5) also known as “The Nelson Mandela Rules;” Convention against Torture and Other Cruel, Inhuman or Degrading Treatment or Punishment (6); Convention on the Rights of Persons with Disabilities (7); Council of Europe European Prison Rules (8)] have helped to provide levers to improve care, as has litigation arising from failures of service provision in some jurisdictions (9) (see for instance Brown v. Plata, 563 U.S. 493, 2011). Despite this, actual service delivery and quality of care delivered has generally remained inadequate to level of need (4, 10–13).

The key elements of correctional mental health services (CMHS) have been articulated for over 30 years. These elements are proactive case detection and assessment, offering a suitable range of services and reintegration planning (14, 15). Steadman et al. (16) first described the need to focus on multiple potential points of engagement or diversion for people with SMI in interaction with the criminal justice system noting key intervention points as being at police arrest, court appearance, remand prison and sentenced prison levels, including re-entry and probation/parole level in the community. This gave rise to conceptual models built along this journey, the most prominent being the Critical Time Intervention (CTI) Model of Draine et al. (17) and Draine and Herman (18) which is a framework providing specific time-based interventions to enhance supports and service provision at key points along this pathway. More recently, Forrester and Hopkin (19) have reviewed CMHS from the perspective of defining these service elements as part of a care pathway. This concept of a pathway or a trajectory for people with SMI is now common (20).

There have been three studies of an overall pathway of care for persons with SMI in correctional facilities in a single jurisdiction (21–23). These studies demonstrate the need for frameworks to address the core service quality issues in correctional mental health care, namely access rates, nature and quality of services delivered, resourcing of clinical teams and management of progression, most particularly between institution and at the point of release.

From work in the UK (1, 24), New Zealand (23, 25), Canada (4, 15), and Ireland (22) and building on the key elements of CHMS previously articulated, there emerged a consensus around the fundamental elements needed for service delivery in custody. We coined the acronym “STAIR” Model (1, 26) to define these elements. STAIR stands for Screening, Triage, Assessment, Intervention, and Re-Integration. The STAIR model also links key clinical functions to epidemiologically derived access and intervention targets, providing benchmarks by which to measure performance. Briefly, the model is as follows.

Screening should be available for all inmates at the point of reception, performed by health staff.

The major disorders being screened for are illnesses such as psychosis, major mood disorders, active suicidality or withdrawal from alcohol or other substances. The rate of positive screens is commonly over 30% of remand men and near 50% of remand women (27) allowing a clinical service to evaluate whether the expected rate of positive screen is being achieved.

**Triage**. Most current screening tools have high false positive rates, so a second stage of evaluation by mental health staff is required, referred to as triage. This is a more detailed assessment of the individual's mental health needs and current level of functioning allowing referral to a next level of primary or secondary care.

**Assessment**. Positive triage will lead to evaluation by a specialist mental health team, including psychiatric assessment and the development of an individual care plan. It should result in ~15% of the standing prison population being attached to a specialist mental health team (2).

**Intervention**. A comprehensive range of culturally competent mental health services is required to respond effectively to the differential levels of presenting illness acuity (e.g., acute or intermediate care for those who are severely or acutely unwell, are suicidal or general prison mental health services for those with more stable conditions).

**Re-integration**. Planning for community reintegration should begin well in advance of the identified release date, to ensure the continuous delivery of healthcare services and that social care services are engaged. This package of care includes engagement with community mental health services and addressing unmet needs in respect of housing, employment and finance. The provision of transitional clinical support during the period of institutional release is preferred.

The purpose of this paper is to review the extant CMHS literature to assess the current evidence in relation to each of these core service elements. We undertook a systematic review of published review articles of each of the service components of the STAIR model. We aimed to describe the current state of knowledge, highlight areas of good quality evidence and identify gaps in knowledge to inform future research.

## Methods

### Search Strategy

We performed a systematic review of reviews adhering to PRISMA guidelines as well as those laid out in the Joanna Briggs Institute Manual for Evidence Synthesis (28). Three separate searches were conducted for each of the following STAIR elements: (1) screening, triage, assessment (grouped together given that similar tools are used across these stages), (2) intervention, and (3) reintegration. Search terms were used to specify setting (correctional settings), population (severe mental illness), and study type (review paper) across all searches. Each of the three searches were conducted in MedLine and CINAHL. Each database search employed search terms describing (1) the STAIR component under investigation; (2) the setting (correctional); (3) population specifiers (severe mental health-related); and (4) specifiers for article type (reviews). These were combined using “AND” statements and each search was assessed for completeness using a set of pre-selected validation articles. The search was limited to review articles published in English from 1995 up until the search date (end of January, 2020) with no date or geographical restrictions.

The search was supplemented in several ways, given that some expected literature may not be indexed in MedLine or CINAHL. To this end, we also searched the Web of Science Core Collection, the Web of Science Conference Proceedings Index, Worldcat/OAlster, and searched government and non-governmental organization websites. Each of these searches used a condensed set of the terms (the search strategy is attached as Supplementary Materials).

### Inclusion Criteria

**Figure 1:**
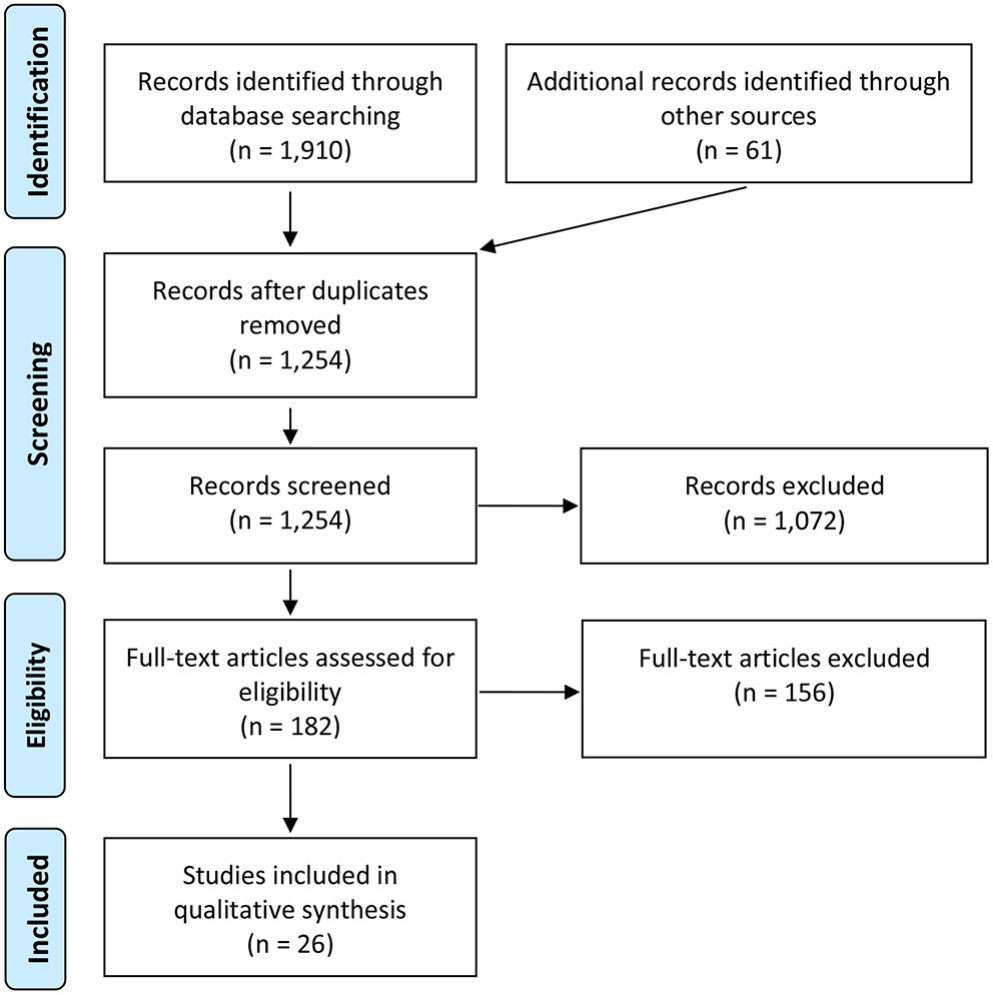
PRISMA flow chart.

We included reviews exploring core CMHS service elements; involving adult prisoners or jail populations with SMI (i.e., psychotic disorder, bipolar disorder, and current severe depressive disorder). Our outcomes of interest were improvement of mental health outcomes broadly (identifying need, reducing symptoms, improving functioning or well-being, accurately identifying SMI). We also applied these criteria to the supplementary searches, except that these were restricted to material that reported data (i.e., opinion papers and unsupported program descriptions were excluded). We excluded papers that (1) provided general discussion or recommendations of services without a review component, or (2) focused only on criminological (e.g., antisocial behavior, recidivism) outcomes among SMI inmates, or outcomes related to suicide or self-harm without specific reference to SMI outcomes, or (3) only focused on substance use or sex offending. Papers with outcome measures that overlapped with those listed above were not excluded. Refer to Figure 1 for detailed PRISMA flow chart of the identification, screening, eligibility assessment, and inclusion of articles.

### Study Selection

Two independent reviewers performed title and abstract screening and, where disagreements existed, a third reviewer arbitrated the decision. Finally, the first author (AS) reviewed the selected articles to exclude those that were superseded by a more recent, more comprehensive or higher-quality review in the same content area.

### Data Extraction and Evaluation of Quality

One rater extracted the data into a pre-defined data extraction table (see Table 1), and a second rater confirmed the accuracy of the rating. Any disagreements were resolved by a third rater. The quality of reviews was also evaluated by two raters using the AMSTAR-2 (A MeaSurement Tool used to Assess systematic Reviews; see Table 2) except for non-systematic or narrative reviews which could not meet AMSTAR Criteria (48).

**Table 1:** Data extraction table of included articles.

**Reference**	**Main focus of review paper**	**Elements of STAIR^a^**	**Number of studies in systematic review**	**AMSTAR score^b^**	**Funding reported**
**Systematic Reviews/Meta-analyses** **=** **12**					
Barker et al. (29)	Evidence-based strategies for managing suicidal and self-harm behaviors in prisons	Intervention	12	Critically low	This review was supported by the Queensland Corrective Services.
Deslich (30)	Telepsychiatry in correctional facilities improves access to mental health care and costs	Intervention	49	Critically low	None stated
Fontanarosa et al. (31)	Evidence for treatments for offenders with serious mental illness in jail, prison, or forensic hospital, and transitioning from any of these settings to the community	Intervention Re-integration	19 papers describing 16 studies	High	None stated
Hopkin et al. (32)	Interventions for prisoners with mental health conditions that target transition from prison to community	Re-integration	14	Moderate	Self-funded
Kendall et al. (33)	Findings from qualitative evaluations of community re-entry programs	Re-integration	8	Moderate	Health Futures Development Grant from the University of Technology Sydney
Martin et al. (27)	Compared the sensitivity and specificity of mental health screening tools among adult jail or prison populations	Screening Triage Assessment	24	Moderate	None stated
Maruca and Shelton (34)	Summarizes correctional nursing interventions for incarcerated persons with mental disorders	Intervention	16	Low	None stated
Morgan et al. (35)	Treatment effects across studies from service providers to offenders with mental illness	Intervention	8	Critically low	None stated
Moyes et al. (36)	How prison-based services can improve to better meet the needs of prisoners with co-occurring substance misuse and mental health disorders	Intervention	67	Critically low	None stated
NICE (37)	This guideline was developed to advise on identification and management of mental health problems and integration of care for adults in contact with the criminal justice system	Screening Triage Assessment Integration Re-integration		High	NICE
Smith-Merry et al. (38)	Brings together existing evidence to inform policymakers and practitioners about current practices in transition support, and barriers and facilitators of effective practice	Re-integration	23	Low	Inner West Partners in Recovery Flexible funding
Yoon et al. (39)	Systematically reviews psychological therapies with mental health outcomes in prisoners and qualitatively summarize difficulties in conducting randomized clinical trials (RCTs)	Intervention	27	Moderate	Wellcome Trust (202836/Z/16/Z)
**Narrative Reviews** **=** **14**					
Baillargeon et al. (40)	Reviews challenges to community re-integration among mentally ill prison inmates and promising strategies for improving transition from prison to community	Re-integration		NA	None stated
Draine and Herman (18)	Reviews the utility of the Critical Time Intervention (CTI) model, and how to assess its effectiveness	Screening Triage Assessment Integration Re-integration		NA	National Institute of Mental Health (NIMH)
Draine et al. (41)	Reviews the utility of the Critical Time Intervention (CTI) model, and relevant background research on re-entry and integration	Re-integration		NA	National Institute of Mental Health (NIMH)
Edens et al. (42)	Review of dual diagnosis treatment programs developed for state and federal prisons in the U.S.	Screening Triage Assessment Intervention Re-integration		NA	None stated
Fazel et al. (3)	Review of clinical, research, and policy recommendations to improve mental health care in prisons	Intervention		NA	None stated
Forrester and Hopkin (19)	Review the nature and extent of evidence streams supporting health care delivery within interagency pathway developments	Screening Triage Assessment Intervention Re-integration		NA	None stated
Forrester et al. (1)	Reviews issues related to service provision of mental health care in prisons and jails and proposes the utility of the STAIR model	Screening Triage Assessment Intervention Re-integration		NA	None stated
Jemelka et al. (43)	Reviews the issue of mental illness in jails and prisons; Includes some treatment and reintegration practices in the U.S. as well as recommendations	Screening Triage Assessment Intervention Re-integration		NA	National Institute of Justice
Kolodziejczak and Sinclair (44)	Reviews a brief history and overview of mental health services in the U.S. correctional system, as well as a discussion of the barriers to and potential facilitators of providing effective care in the future	Screening Triage Assessment Intervention Re-integration		NA	None stated
Ogloff (15)	An overview of Canadian-developed correctional and forensic mental health services to identify and accommodate the needs of mentally ill people in the criminal justice system. A six-component model for mental health services in corrections is advocated in this report. Covers related issues of diversion from jails and the need for suicide risk identification and management in jails.	Screening Triage Assessment Intervention Re-integration		NA	None stated
Peters et al. (45)	Review of the existing research, examination of key issues and evidence-based treatment, and supervision practices related to co-occurring mental and substance use disorders in the justice system	Screening Triage Assessment Intervention Re-integration		NA	None stated
Simpson et al. (4)	Reviews the required service components with particular focus on care models for people with serious mental illness in the Canadian correctional system	Screening Triage Assessment Intervention Re-integration		NA	None stated
Wallace et al. (46)	Provides evidence-based and promising treatment approaches to address the overlap among trauma, mental illness, substance abuse, and behavioral problems. A synthesis of research meant to guide practitioner and policy responses to the national challenge of meeting the needs of those undergoing re-entry	Re-integration		NA	National Institutes of Health (NIH)
Winters et al. (47)	Reviews interventions designed to prevent suicide among individuals with serious mental illness in forensic settings, and the need for research to inform the development of assessment tools and intervention strategies for this population	Screening Triage Assessment Intervention		NA	None stated

a *STA, Screening, Triage, and Assessment; I, Intervention; R, Re-integration; MoC, Model of Care*.

b *NA, Not applicable; narrative review articles that were not graded with AMSTAR*.

**Table 2:** AMSTAR-2 ratings for included systematic reviews and meta-analyses.

**References**	**AMSTAR questions^a^**																**Overall confidence**
	**1**	**2**	**3**	**4**	**5**	**6**	**7**	**8**	**9**	**10**	**11**	**12**	**13**	**14**	**15**	**16**	
Barker et al. (29)	Y	N	Y	PY	N	N	N	Y	N	N	NM	NM	N	Y	NM	N	Critically low
Deslich (30)	Y	N	N	PY	N	N	N	PY	N	N	NM	NM	N	Y	NM	Y	Critically low
Fontanarosa et al. (31)	Y	PY	Y	Y	Y	Y	Y	Y	Y	Y	NM	NM	Y	Y	NM	Y	High
Hopkin et al. (32)	Y	PY	Y	Y	Y	N	PY	PY	Y	Y	NM	NM	N	Y	NM	Y	Moderate
Kendall et al. (33)	Y	N	Y	PY	N	Y	Y	Y	N	N	NM	NM	N	Y	NM	Y	Moderate
Martin et al. (27)	Y	N	Y	PY	N	Y	PY	PY	Y	N	NM	NM	N	N	NM	Y	Moderate
Maruca and Sheldon (34)	Y	PY	Y	PY	N	N	N	Y	Y	N	NM	NM	Y	Y	NM	Y	Low
Morgan et al. (35)	Y	N	N	PY	N	Y	N	Y	Y	N	Y	N	Y	N	Y	N	Critically low
Moyes et al. (36)	Y	N	N	N	N	N	N	N	N	N	NM	NM	N	N	NM	N	Critically low
NICE (37)	Y	Y	Y	Y	Y	N	Y	Y	Y	N	Y	Y	Y	Y	Y	Y	High
Smith-Merry et al. (38)	Y	N	Y	PY	Y	N	N	N	N	N	NM	NM	N	Y	NM	Y	Low
Yoon et al. (39)	Y	Y	Y	PY	N	Y	PY	Y	Y	N	Y	Y	Y	Y	Y	N	Moderate

*Y, Yes; PY, Partial Yes; N, No; NM, No meta-analysis conducted*.

a *1. Did the research questions and inclusion criteria for the review include the components of PICO? 2. Did the report of the review contain an explicit statement that the review methods were established prior to the conduct of the review and did the report justify any significant deviations from the protocol? 3. Did the review authors explain their selection of the study designs for inclusion in the review? 4. Did the review authors use a comprehensive literature search strategy? 5. Did the review authors perform study selection in duplicate? 6. Did the review authors perform data extraction in duplicate? 7. Did the review authors provide a list of excluded studies and justify the exclusions? 8. Did the review authors describe the included studies in adequate detail? 9. Did the review authors use a satisfactory technique for assessing the risk of bias (RoB) in individual studies that were included in the review? 10. Did the review authors report on the sources of funding for the studies included in the review? 11. If meta-analysis was performed, did the review authors use appropriate methods for statistical combination of results? 12. If meta-analysis was performed, did the review authors assess the potential impact of RoB in individual studies on the results of the meta-analysis or other evidence synthesis? 13. Did the review authors account for RoB in primary studies when interpreting/discussing the results of the review? 14. Did the review authors provide a satisfactory explanation for, and discussion of, any heterogeneity observed in the results of the review? 15. If they performed quantitative synthesis did the review authors carry out an adequate investigation of publication bias (small study bias) a discuss its likely impact on the results of the review? 16. Did the review authors report any potential sources of conflict of interest, including any funding they received for conducting the review*.

### Data Synthesis

Narrative data synthesis was performed by examining the characteristics and findings of the included reviews, and summarized in the data extraction table. The breadth, quality and consistency of reviewed materials reported were considered in relation to the quality ratings of each review (AMSTAR), and according to clinical and research considerations as adjudged by the current study's authors. Key information pertaining to main findings, study methodology, gaps and future directions were highlighted.

## Results

We identified 26 reviews that met inclusion criteria for the study (see Table 1 for a summary). Seven reviews referenced the need for an integrating model of care as a concept, but there were only two papers that provided primary data regarding models of care (22, 23) leaving insufficient comparative data to review overall care pathways.

### Screening, Triage, and Assessment: General Overviews and Care Pathways

Our search found two systematic reviews (27, 37) and seven narrative reviews that discussed screening and assessment processes. The methodological quality of the systematic reviews was assessed by AMSTAR 2 and presented in Table 2.

Forrester and Hopkin (19) reviewed pathway models across the criminal justice system with a focus on developments since 2000. Part of their review concerned care in corrections, although they primarily referred to the systematic review of Martin et al. (27). They note that while screening has been a research focus in this area, additional work is needed to ensure coverage of the broad range of disorders seen in correctional settings. The major issue that concerns all individual screening tools is that of poor specificity or the problem of high false positive rates (3). In their narrative review, Kolodziejczak and Sinclair (44) emphasize the need to strike a balance between risks related to over-diagnosis and risks related to under-diagnosis. They note that while under-diagnosis in corrections has clear negative impacts given evidence that many persons with SMI do not access treatment, over-diagnosis has implications for stretching limited health resources which may result in under-attending to those with the most severe needs. Others (49, 50) have recommended a two-tiered screening process using a highly sensitive screen on admission to ensure that those in immediate need (including those with SMI, or at high risk for self-injury or suicide) are attended to without delay followed by a later, more comprehensive and specific stage that can sort those positive screens into those who do and do not need further mental health assessment. This is essentially the logic of the “ST” component of STAIR (1).

#### Special Considerations for Comorbid Substance Use in SMI

Some reviews had a focus on those inmates with comorbid substance use and mental health diagnoses (i.e., concurrent disorders). While the focus of the Moyes et al. (36) review was on treatment, they noted that screening for concurrent disorders was lacking in many facilities. They recommended the integration of concurrent disorder assessment into existing, post-admission visits made by in-reach teams (i.e., during the “T” or “A” components of STAIR), given the challenges of performing such assessments upon admission. In contrast, Peters et al. (45) recommended that concurrent disorders be screened at admission, as well as at several other junctures, including initial probation/parole and re-entry points. They note that there are few screening measures that simultaneously address SMI and substance use disorders, and recommend the use of a combination of tools to ensure coverage of both domains.

#### Screening, Triage, and Assessment: Specific Tools

Martin et al. (27) performed the seminal systematic review and meta-analysis of screening and triage tools in prison. They identified three screening tools and one triage tool that had robust developmental data and independent validation.

#### Correctional Mental Health Screen for Women and Correctional Mental Health Screen for Men

The CMHS-W and CHMS-M are gender-specific tools containing eight and 12 staff-administered items, respectively. Martin et al. (27) cite two studies meeting inclusion criteria; the development paper (51) and a replication by the same authors (52). For the CMHS-W, they found consistent sensitivity rates between the two studies (65 and 64%, respectively). For the CMHS-M, somewhat higher, similarly consistent sensitivity rates were found between the development and replication studies (74 and 70%, respectively). These two tools have been replicated in a separate sample performed by the original authors with highly consistent findings. QUADAS assessment (26) identified high risk of bias in both of the Ford et al. (51, 52) studies with regard to index test use, and applicability concerns in Ford et al. (51) with regard to index test use. The more recent NICE Guideline on the Mental Health of Adults in Contact with the Criminal Justice System (37) did not uncover additional studies on these measures up to 2016.

#### Brief Jail Mental Health Screen

The BJMHS (53) is a widely-used staff-rated screening tool. It consists of six symptom-related items and two additional items related to medication and hospitalization. Martin et al. (27) found the original Steadman et al. (53) development article and four additional validation articles; one by the scale's authors (54) and three independent studies. The BJMHS was found to show sensitivity of ~60–65%, with the notable exception of one study (55) that yielded a sensitivity of only 34% among male inmates. When used to screen female inmates, it was found to yield lower sensitivity [e.g., 46% per Steadman et al. (53)] and may not be considered well-validated for female inmates [as noted in Kubiak et al. (56)]. With regard to rigor, QUADAS ratings were generally positive, with no concerns noted for the Evans et al. (55) study. For three of the studies reviewed (53, 54, 57), they note risk of bias in patient selection (in addition to risk related to flow and timing in the latter paper), and with regard to Ford et al. (51) they note risk of bias related to the index test used. NICE (37) revealed one additional study (58) yielding an Area Under the Curve (AUC) of 0.72 in a police jail context. NICE appraised the risk of bias in this paper to be high in terms of index test use and reference standard.

#### England Mental Health Screen

The EMHS (59) is a four-item tool with a yes/no format, with items focusing on historical factors. A single “yes” response constitutes a positive screen. Martin et al. (27) found four studies including a small pilot, two follow-up studies by teams including the scale's primary author, and one independent study (55). They note that the scale achieved 100% sensitivity in the pilot (59) but in validation studies involving all-male populations, achieved sensitivities of 42 and 76% (55, 60) In terms of rigor, QUADAS ratings revealed low risk of bias in the original pilot and Evans et al.'s (55) validation, but raised concerns regarding index test selection in the Birmingham et al. (60) study and multiple issues with a study by Gavin et al. (61). The inconsistency in findings across available studies give rise to caution and the potential importance of moderating variables.

#### Jail Screening Assessment Tool

The JSAT (15) is a structured professional judgment guide and, as such, requires expertise to administer, having the characteristics of a triage tool, in terms of the STAIR model. It is a semi-structured interview lasting ~20 min. It reviews a broad range of factors including mental health issues, current symptoms, substance use, social support, legal situation, and violence. Martin et al. (27) reviewed the original development study (15, 62) and two independent validations (57, 63). Among males, JSAT showed a sensitivity between 38 and 84%, with the latter figure coming from the development study. Among women, it achieved a sensitivity of 75%. Martin et al. (27) highlighted the wide range of findings and interpret this as stemming from the manner in which the JSAT employs structured professional judgment. When a structured scoring cut-off was proposed in one study (57), it yielded a sensitivity of 67–72%. In terms of the rigor of the reviewed studies, QUADAS ratings reflect concerns with bias stemming from patient selection in all studies.

### Interventions

We found eight peer reviewed systematic reviews and seven narrative reviews that discussed interventions within correctional institutions. One gray literature systematic review (37) met our inclusion criteria. The methodological quality of the systematic reviews was assessed by AMSTAR 2 and presented in Table 2.

Kolodziejczak and Sinclair (44) in their narrative review concluded that there is a lack of interventions proven effective for SMI typically available in prisons and noted that, when mental health services are received, they may be limited to medication management due to high caseloads. They nonetheless noted the effectiveness of combined pharmacological and psychotherapeutic approaches, and stressed the importance of addressing comorbid substance use and SMI. They concluded that very little literature specifically evaluates the treatment of SMI within correctional facilities, due to a number of barriers and limitations. Fazel et al. (3) likewise concluded that few studies exist in this area, and those that do tend to be small and yield inconsistent results. A paucity of pharmacological studies was specifically noted.

Yoon et al. (39) conducted an extensive review and meta-analysis of RCTs for psychological interventions in corrections and found a moderate overall effect size of *d* = 0.50 across interventions, outcomes and comparators, albeit with large heterogeneity. No difference was found between group and individual administration, but the authors cautioned against assuming equivalence given differences in mean duration between these modalities. Their review yielded seven RCTs with high quality ratings (among the 37 assessed) and found specific support for mindfulness-based and CBT-based interventions, especially for treating depression and anxiety. Martin et al. (64) also conducted a large meta-analysis of interventions designed to reduce criminality or improve mental health in inmates with SMI. They analyzed 25 studies with various modalities, comparators and treatment goals and found evidence for reduced recidivism, better functioning and reduced symptoms across studies. High attrition/rapid turnover, small samples, difficulties in implementing manualized treatments, and loss of effect at follow-up time points were commonly identified.

Some reviews focused on specific treatment modalities, as outlined next.

#### Pharmacotherapy

Fazel et al. (3) and Fontanarosa et al. (31) found very few trials for pharmacotherapy in correctional settings, relative to psychological interventions. Fazel et al. (3) included only two, including a trial of ADHD medication improving functioning and promoting abstinence from amphetamine use post-release (65, 66) and a trial for a pharmacotherapy decision-making algorithm that resulted in a null finding (67). Fontanarosa et al. (31) reported that evidence is lacking to draw any strong conclusions regarding pharmacotherapy interventions specific to correctional settings; these authors limited their review to trials with active control arms.

#### Cognitive-Behavioral Therapy

CBT was, across reviews, the most widely-studied form of psychotherapy in correctional settings. This category included reviewed studies of standard CBT as well as interventions employing CBT principles. Yoon et al. (39) performed the most exhaustive review of CBT among the studies reviewed, and examined CBT separately in their meta-analysis. They found 14 RCTs of CBT with a variety of outcome measures and control groups, and concluded that there is moderate-quality evidence supporting this treatment, particularly for anxiety and depression. They did not find evidence supporting the superiority of CBT over other modalities.

#### Dialectical Behavioral Therapy

DBT is a highly structured intervention that includes individual psychotherapy (normally 12 months or more), concurrent skills training groups, and structured consult groups for practitioners. Given the challenges of implementing the full DBT model in correctional settings, it is often implemented in an abridged format, and its primary goal has often been the reduction of aggressive incidents (68). Yoon et al. (39) reviewed one RCT with an adequate quality rating, finding a positive but null effect of DBT on trauma and depression symptoms (69). Fazel et al. (3) reviewed a single RCT of DBT for incarcerated women (compared with a shorter-duration DBT regimen plus case management) and found that the former group showed reduced psychopathology.

#### Interpersonal Therapy

The reviews by Yoon et al. (39) and NICE (37) uncovered only one RCT of ITP (70). NICE concluded that it provided very low-quality evidence for a clinically significant treatment effect in depression.

#### Meditation-Based Interventions

Several current psychotherapies incorporate meditation techniques, such as mindfulness. This category considered approaches based primarily on meditation, including mindfulness-based interventions and Yoga-based interventions. Yoon et al. (39) uncovered five studies in four separate papers examining mindfulness-based interventions in correctional settings, all with risk of bias adequately addressed. They concluded that these therapies were beneficial for symptoms of depression and anxiety. Fazel et al. (3) uncovered one additional, large RCT of a Yoga-based intervention that yielded lower distress and improvements in cognitive function in a prison setting (70). In their review of nursing interventions, Maruca and Shelton (34) additionally found one feasibility study (71) supporting Yoga as a potential treatment for stress and anxiety in incarcerated women.

#### Trauma-Based/Trauma-Informed Interventions

Yoon et al. (39) in their systematic review of trauma informed interventions in corrections found six RCTs of therapies classified as trauma-related (including one additional study of cognitive processing therapy, a CBT-based PTSD treatment). Together, the six RCTs failed to achieve statistical significance in meta-analysis. Individual trials that did yield significant effects vs. waitlist or no-treatment controls included Trauma Incident Reduction Therapy (72) Trauma Recovery and Empowerment Model (TREM) for male inmates (73) a brief trauma group (74) and a DBT-based group (69). Two therapies that did not achieve statistical significance were compared to active therapy. NICE (37) reviewed a subset of the same studies; they rated the evidence stemming from the non-null trials reviewed as being of very low to low quality.

#### Arts-Based Interventions

NICE (37) reviewed one large RCT of arts-based therapy, yielding very low-quality evidence of clinically significant impact on depression (75). Yoon et al. (39) included this study and three additional trials of art- and music-based therapies, and found that two trials of art-based therapies vs. no treatment, and one out of two trials of music-based therapy vs. an active comparator, yielded positive effects.

#### Telehealth Interventions

Deslich (30) reviewed the implementation of telepsychiatry services in correctional settings (vs. in-person services) and found that although telehealth is a platform rather than an intervention, these services improve access without appearing to negatively impact inmate experiences of care, while significantly reducing costs. Fontanarosa et al. (31) cite a prior review by Khalifa et al. (76) suggesting effectiveness of telepsychiatry across multiple forensic settings, including prisons, but note limited outcome-related evidence in this area.

#### Substance Use and Concurrent Disorders

Multiple reviews noted the particularly high rate of substance use disorders in those with SMI in incarcerated populations [e.g., up to 80% (2)], and the importance of simultaneously treating both disorders as per the Integrated Dual Diagnosis Treatment (IDDT) model. This broad framework treats substance and mental health disorders together rather than in parallel or serially, often incorporates intervention models such as CBT and therapeutic community approaches, and yields outcomes superior to approaches targeting either disorder category alone or in parallel (36, 45). In their narrative review, Peters et al. (45) noted that given the relatively short time frame of admission to jails, focus should be on acute care, withdrawal management, and community linkage. They found very few studies of in-jail programs and these tended to be non-integrated and lacking in quantitative data. In terms of prison settings, they found that therapeutic communities (TCs: see the subsection below) had support in comparison with other mental-health focused programming in terms of long-term impact on relapse and re-arrest. Some additional recommendations in this area included the future collection of better-quality evidence, tailoring treatments to gender and stage of change, using peer mentorship, minimizing confrontation and addressing criminogenic thinking (36, 42, 45).

NICE (37) evaluated several other approaches to substance use disorders, including psychological (e.g., CBT and Acceptance and Commitment Therapy) and pharmacological (e.g., Naltrexone and methadone maintenance) approaches. The majority of these were not specific to SMI populations and examined only substance-related and legal outcomes. The evidence for psychological approaches was of very low to low quality, primarily used active psychological comparison groups and revealed predominantly null findings. They notably examined several RCTs of Naltrexone vs. placebo and found very low-quality evidence of opioid use reduction with Naltrexone treatment.

#### Therapeutic Communities

TCs are milieu-based, interdisciplinary, multifaceted approaches to treating substance use disorders, often incorporating cognitive and behavioral components. Fontanarosa et al. (31) concluded that there is insufficient evidence to judge the comparative effectiveness of TCs and traditional in-prison care for comorbid conditions. NICE (37) uncovered eight RCTs examining TCs and Modified Therapeutic Communities (MTCs) in corrections, yielding very low to low quality evidence for efficacy on a number of psychological symptom and substance use-related indicators, including improvements in substance use for MTCs vs. a CBT-informed group and vs. a traditional mental health program, and mood improvements in an MTC vs. a TC. Several comparisons between TCs, MTCs, and other active control arms in this review were null and considered of very low quality.

#### Suicidality Interventions

Winters et al. (47) conducted a review of suicide prevention strategies in SMI populations in corrections and noted that, while CBT, DBT, and IPT programs have shown efficacy in preventing suicide in general settings, these are difficult to implement in corrections, and sparse research exists on corrections-specific programs. They did not find any corrections-specific literature on pharmacological interventions. Barker et al. (29) performed a systematic review of effectiveness literature on suicide and self-harm prevention strategies in prisons, which yielded 12 relevant studies. These were predominantly program implementation studies with AB designs, and included improved assessment and monitoring, training (notably including the training of peer supporters), special focus on SMI populations and inmates with borderline personality disorder, and review/debriefing strategies. They concluded that such multi-factored interventions focused on mitigating risk factors are particularly effective in reducing suicide outcomes across reviewed studies.

### Reintegration

Reintegration programs focus on the transition period for inmates with SMI who are being released from custody to ensure continuity of their mental health care and other social needs. We found three systematic reviews and six narrative reviews of interventions aimed at transitioning individuals with SMI from custody. Additionally, three gray literature articles met inclusion criteria (see Table 1). The majority of reintegration programs reviewed were from the United States (37) and targeted both the pre- and post-release periods though the actual length of the programs varied widely (31, 32, 38). The results of the AMSTAR 2 quality assessments of the included reviews are presented in Table 2.

Certain common features of reintegration programs were pre-release planning and post-release support with a combination of practical resources and empathic support (32, 33, 40). These supports can be through remote follow-up or in-person engagement to assist patients having trouble navigating the system (32) and linking them with appropriate community case management (40). This requires trained staff with knowledge of community services. Individualized assessment with a written release plan of the needs and the public safety risk of the inmate (40) are also crucial to avoiding gaps in treatment. Different approaches are required for remand or pre-trial populations due to the shorter term stays and more unpredictable discharge requiring the assessment of needs to be fast-tracked (17). Traditionally, the goal of re-entry has been to reintegrate the individual into the community with the focus of protecting the community from future harm (41) as opposed to the recovery-oriented and patient centered care that is now the industry standard for mental health services generally (38).

Outcome measures commonly employed included health outcomes such as service use, hospitalizations and medication adherence, and criminal justice outcomes such as reoffending and reincarceration. Severity of symptoms of SMI were rarely used as an outcome measure. Only four of the reviews employed evidence quality assessments in their review methodology (31–33, 37). Lack of blinding was the biggest issue for weak studies (32).

Effectiveness of programs using criminal justice outcomes was assessed in several studies with only one reporting a significant reduction in reoffending and reincarceration (77) though the evidence was weak due to factors including selection bias and confounders. The evidence for research on other programs were rated of low to high quality. The two studies rated as high reported a non-significant reduction in re-arrests (78) and an increase in reincarceration, respectively (79). Hopkin et al. (32) posited that the increased monitoring offered by the reintegration program may serve as a possible explanation for the increased reincarceration. Studies assessing mental health outcomes were also reported to be of varying quality. IDDT programs that reported reduced psychiatric hospitalizations and mental health service use were of low quality (31, 37) with insufficient evidence for impact on substance abuse (31). Research on other interventions reported significantly higher mental health service contacts than the comparator groups and were assessed by Hopkin et al. (32) to be of moderate to high quality.

Three of the trials reviewed by Fontanarosa et al. (31) were conducted in urban areas making it not transferrable to rural areas where community resources may be scarce. Evidence for the impact of specialist vs. mental health generalist care on psychiatric symptoms, psychiatric hospitalization, substance abuse, quality of life, and completed suicide was insufficient as only one trial reported these outcomes (31). The same authors also reported an RCT on Interpersonal therapy (IPT) demonstrating reduction in depressive symptoms but no change to substance-abuse relapse with low risk of bias though this was on the only study on this program and thus insufficient to draw conclusions. A more recent qualitative study of this program also reported program satisfaction with high quality (33).

Multiple barriers to reintegration have been described including lack of funding (38, 40) complex post-release care pathways, the need for greater direct service connectivity, insufficient planning resources, a lack of collaboration between correctional facilities and the community and unavailability of medication at release (38). Additionally, the chaotic nature of release particularly for remand inmates may limit the ability of community services to respond to referrals (19). Programs including CTI (80) may not be feasible in rural or regional settings where community mental health resources are scarce (38). Unconditional releases (without parole and mandated treatment) pose the most difficult challenges with transitional planning (40). This may be due to difficulties accessing inmates for their participation in re-entry support and lack of participation may reflect concerns and motivations that are independent of the need or desire for mental health care (38).

The majority of reintegration programs fell into the following categories:

1. Bridging plus assistance with benefits application: Programs reviewed were specific to the US where 90% of jurisdictions terminate or suspend Medicaid upon incarceration and lack of affordable healthcare may mean that many inmates need benefits to continue accessing care upon release (38). The bureaucracy involved in reinstating benefits may impede those with SMI. Transition planning teams have been shown to improve post-release benefit enrolment (40) but the impact on improving mental health outcomes is unclear with limited evidence reported for future contact with the mental health system (31, 32, 38).
2. ACT programs: Adapted ACT programs ensuring ongoing care for individuals leaving custody is common. One RCT measuring psychological and clinical outcomes demonstrated no significant difference between ACT, forensic caseworkers and treatment as usual (32). Another program used an ACT model to pair probation officers with mental health workers for persons with comorbid SMI and substance use showing less likelihood of re-incarceration though these results were not significant (32, 78).
3. Critical time intervention (CTI) and short-term bridging: CTIs for transition support are focused, time-limited interventions that aim to develop an individualized housing, education and employment strategy to increase social inclusion. Such programs are designed to be short-term and connect individuals with community care (18, 38). These programs are less effective in areas where community resources are scarce and not feasible unless the case manager is located in the correctional facility (38). In the UK, an RCT on CTI (4 weeks pre and 6 weeks post-release) demonstrated significantly higher registration with a general practitioner (87 vs. 38%; *p* = 0.01) and medication administration (80 vs. 38%; *p* = 0.03) although the results lack sufficient power due to the high attrition rate (19, 32). A larger RCT by Shaw et al. (81) found that CTI significantly improved engagement with community mental health services at 6 weeks (53 vs. 27%, *p* = 0.012) and this was maintained at a later follow up 6 months (*p* = 0.029) after release (19, 32).

With regard to co-occurring substance use, while some reintegration programs addressed substance use together with SMI (32) re-entry services were often fragmented and were only focused on mental health issues and not sufficient to address other risk factors for criminal recidivism which may not be a result of mental health symptoms (45). Advances have been made in co-occurring disorders (CODs) treatment but such programs are still absent in many communities and correctional facilities (45). IDDT programs shows promise for reducing hospitalization post-release but replication studies are needed (31). Services integrating mental health and substance misuse services should be delivered by staff who have expertise in both areas rather than sequentially or in parallel (45). There are few studies on CODs programs targeted toward female offenders (45). In their review of qualitative studies, Kendall et al. (33) reviewed one such study on female inmates with SMI noting that women valued continuity of care with the same worker.

## Discussion

The needs of persons with SMI in correctional settings remain of major concern. In this review of the reviews of correctional mental health care elements, we set out to describe the state of knowledge of the span of the care pathway during incarceration. To do this we used the organizing structure of the STAIR model to define the key service domains of this care trajectory to enable us to evaluate the strength of knowledge at each step.

We found a very significant number of reviews. However, many were narrative in form and, whilst informative and containing much wisdom about the development and implementation of correctional mental health services, are limited in their generalizability because of the lack of empirical studies upon which to base their guidance. We found 12 systematic reviews or meta-analyses that focused on the domains of screening, interventions and re-entry programs. The areas of greatest knowledge are in screening and triage, psychological therapies and aspects of reintegration in certain jurisdictions.

In the screening and triage area, there are two high quality systematic reviews of multiple tools with independent validation studies. This evidence is sufficient to make recommendations for service design using two screening tools of adequate psychometric integrity (BJMHS and CMHS) and one triage tool (JSAT). Both independently validated screening tools have problems of high false positive rates necessitating triage processes if they are employed in settings with large numbers of persons to be screened. The JSAT is the only validated longer form assessment tool that may be appropriate for the triage of persons referred on the basis of shorter tools such as the BJMHS or CMHS. Proper staff training in the JSAT is crucial given evidence for wide variability in performance across settings in this largely subjectively-rated instrument, while screening tools offer more consistent results and can be administered by non-specialists. These tools remain poorly validated for women, and for those of minority ethnicity. All of the reviews in the S-T-A area focused on measures that are typically implemented in the S and T stages of STAIR. There is a lack of evidence concerning in-depth assessment tools and processes in these populations.

There were eight systematic reviews of interventions, with a sufficient number of robust studies for meta-analyses of some psychosocial interventions. There were few studies of biological interventions in custody. This limited research base in the specific context of correctional facilities may reflect the assumption that the effectiveness of pharmacotherapy interventions for specific disorders are reasonably generalizable from trials of similar patient groups in other settings. The same may well not be true for psychological interventions, which may be more heavily moderated by contextual and population-specific factors, and often require modification in correctional settings. There is thus less need to replicate efficacy studies of psychotropic medications in custody than there is a need to demonstrate the efficacy of psych-social interventions. For instance, an effective intervention such as DBT requires significant modification for correctional settings (67) meaning specific trials are needed to demonstrate effusiveness of the modified intervention specific to the mental health and environmental challenges of living in custody. Study in this area is challenging, given setting- and duration-related restrictions. There is now a solid body of evidence for CBT for anxiety and depressive symptoms for persons in custody, whereas sparse or low-quality evidence supports the efficacy of other modalities and the psychological treatment of other presenting problems. Feasibility studies, on the other hand, appear common in this area and support the application of modified forms of several psychological therapies in corrections. Heterogeneity and inconsistent findings are the norm in this field, suggesting that the examination of modifying factors might be a fruitful avenue for future research. Telehealth also appears to be a promising delivery mode for psychotherapy, with early support for non-inferiority and feasibility; this could reduce access barriers in many correctional settings, including for those in segregation.

Reintegration remains a major transition point where particular models of interventions are required to achieve continuity of care for those with SMI to reduce relapse and recidivism (18). To ensure that help is not misplaced, there is a need for individualized post-release plans to address prisoners' unique needs (38, 40); prisoners may view mental health needs as secondary to economic considerations such as obtaining housing and employment (82). Programs such as Housing First that aim to address inmates' economic needs have showed weak evidence (20).

The body of evidence for reintegration studies is significant but often limited in generalisability because the studies address jurisdictionally-specific issues such as Medicaid enrollment. Though countries such as Canada, the UK, Australia, and New Zealand have public healthcare, inmates may still need support with drug plan applications to ensure continued access to medications such as antipsychotics necessary for managing symptoms and preventing recidivism (83–85). There is a crucial need for more studies addressing comprehensive support models at the point of release that address social determinants of health (benefits, housing) as well as health and criminogenic issues. The problem of rapid re-incarceration of many persons with SMI being released from custody (86) underlines this need. While the purpose of reintegration has shifted from protecting the community from future harm to addressing the inmates' recovery needs, only one study assessed symptom improvement as an outcome (38). Among people with severe mental illness, incarceration is five times more likely among those with a co-occurring substance use disorder (38, 87) yet few re-entry programs were aimed at substance abuse.

Few reintegration studies were specific to women, though research has shown that woman have different demographic, health, and criminal characteristics (3). Factors such as women being more likely to have children will impact their reintegration needs. There were no studies of reintegration of aboriginal populations or other racialized minorities that are overrepresented among incarcerated populations. There was a lack of studies assessing re-entry programs in middle and low income countries despite higher rates of SMI amongst their prison populations (2). Community reintegration programs need effective community mental health care to pick up the care of the person exiting custody. Lower income, marginalized neighborhoods having disproportionately higher numbers of the incarcerated individuals where the scarcity of community mental health resources may result in a cycle of reincarceration (88). Bridging programs may also be particularly challenging in countries that have large regional, rural and remote areas such as Australia (38).

We also found areas of weakness. Whilst the screening tools are well-studied, all have problems with high false positive rates; there are few studies of cross gender effectiveness and cross-cultural effectiveness. Given that persons of minority ethnicity are over-represented in custody, ensuring tools are effective for the particular ethnic groups in a jurisdiction remains a challenge that has been rarely addressed. Second, there are no studies of standardized assessment tools of severity of illness measures in routine use, both to describe need at point of service entry and as measures of effectiveness of interventions or systems of care. The Clinical Global Imopression-Corrections (CGI-C) scale is one promising such tool that has been validated in Canada and Germany (86, 89). There are few studies of the overall care pathway, the studies of O'Neill et al. (22) and Pillai et al. (23) being notable exceptions. More studies of this type are needed linking service provision to quality indicators at multiple points across the care trajectory. The systematic review of intervention studies found too few intervention studies to inform services of effective intervention approaches.

We employed the STAIR model to organize this literature and found it a helpful framework to show areas of strength and areas of weakness in existing research in each area. The principle of seeing CMHS as an integrated care pathway, with measurable levels of access and expected quality outcomes, is crucial to focusing forensic research and delivery initiatives to improve service outcomes.

### Limitations

The major limitation is the diversity of the literature, and too few studies in a number of areas to come to clear recommendations about evidence-based recommendations. As we chose to only review reviews, there may be primary studies in some areas and smaller studies that are in need of replication that we have not included. There may be promising practices in these excluded studies that need to be more rigorously tested in an experimental paradigm.

## Conclusion

There is a rich literature in correctional mental health services with some areas of strength but other areas of weakness. The STAIR model provides a framework to organize our thinking about these needs and to focus more research on care pathways and performance measures. New research is needed into therapeutic interventions and reintegration needs in particular.

## Data Availability Statement

The original contributions presented in the study are included in the article/Supplementary Material, further inquiries can be directed to the corresponding author/s.

## Author Contributions

AS conceived of the review. CG, MM, VA, TV, LF, and TK performed review, the data extraction, and quality ratings. MM, CG, AS, AF, and RJ drafted the manuscript. All authors contributed to the article and approved the submitted version.

## Conflict of Interest

The authors declare that the research was conducted in the absence of any commercial or financial relationships that could be construed as a potential conflict of interest.

## Publisher's Note

All claims expressed in this article are solely those of the authors and do not necessarily represent those of their affiliated organizations, or those of the publisher, the editors and the reviewers. Any product that may be evaluated in this article, or claim that may be made by its manufacturer, is not guaranteed or endorsed by the publisher.
