# Supplementary material for: A Systematic Review of Reviews of Correctional Mental Health Services Using the STAIR Framework
Source: Front Psychiatry. 2022 Jan 18;12:747202. doi: 10.3389/fpsyt.2021.747202 (PMC8806032; doi:10.3389/fpsyt.2021.747202)
Supplement: Supplementary file 1 [file Table_1.DOCX]

**A systematic review of reviews of correctional mental health services using the STAIR framework**

**Database Search Strategy: Summary**

Included databases: Medline, CINAHL

Date limitations: 1995 – end of January, 2020

The search terms below are written in Medline syntax; equivalent searches were constructed for CINAHL

**Screening, Triage, Assessment Search Terms**

| ((assess* or consult* or evaluat* or interview* or inventory or need* or management or measure* or review* or scale* or screen* or structured or tool*) adj3 (assess* or consult* or evaluat* or interview* or inventory or need* or management or measure* or review* or scale* or screen* or tool*)) |
| --- |
| ((assess* or review* or interview or inventory or judgment or measure* or scale* or screen* or tool*) adj3 (accura* or qualit* or perform* or reliab* or valid*)) |
| ((brief or clinical or health or mental or mental health or mental illness or mental disorder or nursing or professional or psychiatr* or psycholog* or strength*) adj4 (assess* or consult* or evaluat* or interview* or judgment or inventory or need* or management or measure* or review* or scale* or screen* or tool*)) |
| ((case or care or individual) adj3 (management* or plan*)) |
| (level adj3 (acuity or acute or security or severe*)) |
| (positive screen or negative screen) |
| (presenting adj3 (issue* or need*)) |
| "Mass Screening"/ |
| (screen* or assess* or triag*) |

**Intervention Search Terms**

| ((acute or affect* or anger or anxiety or art or behavio* or bipolar or borderline or BPD or Brief or CBT or cognitive or community or coping or crisis or DBT or depression or dialetical or early or emotion* or good or group or holistic or humanistic or individual or integrat* or intermediate or MCBT or mindfulness* or meditat* or mood or motivat* or peer* or post traumatic stress or PTSD or prosocial or (psychosis or psychotic) or relapse or resilien* or risk or schizo* or short term or standard or social or solution focused or strength based or stress or substance abuse or substance abuse or telepsych* or trauma or well being) adj3 (care or counsel* or intervention* or maintenance or management or monitor* or need* or practice* or prevention or program* or psychotherap* or recover* or rehab* or service* or support* or therap* or training* or treatment* or workshop*)) |
| --- |
| ((mental disorder* or mental health or mental illness* or psycholog* or psychosocial or psychiatr*) adj3 (care or counsel* or intervention* or maintenance or management or monitor* or need* or practice* or prevention or program* or psychotherap* or recover* or rehab* or service* or support* or therap* or training* or treatment* or workshop*)) |
| ((care or counsel* or intervention* or maintenance or management or monitor* or need* or practice* or prevention or program* or psychotherap* or recover* or rehab* or service* or support* or therap* or training* or treatment* or workshop*) adj3 (adher* or effective* or efficac* or evaluat* or evidence or fidelity or success)) |
| (care or counsel* or intervention* or maintenance or management or monitor* or need* or practice* or prevention or program* or psychotherap* or recover* or rehab* or service* or support* or therap* or training* or treat* or workshop*) |
| (mental disorder* or mental health or mental illness* or psycholog* or psychosocial or psychiatr*) |

**Reintegration Search Terms**

| ((case or client or patient) adj3 (care or management or plan*)) |
| --- |
| ((community or community based) adj3 (accomodat* or access*or aftercare or after care or agenc* or care or coach* or corrections or counsel* or discharge or education* or engagement or follow up or goal* or hous*or integrat* or intervention or life skill* or linkage* or maintenance or manag* or measure* or mental health or monitor* or placement* or prevent* or program* or recover* rehab* or reintegration or re-integration or reentry or re-entry or resettlement or re-settlement or rehab* or resource* or residential facilit* or therap* or service* or supervis* or support* or therap* or triag* or transition* or treat* or workshop*)) |
| (aftercare or after care or in reach or inreach or one stop shop* or one-stop-shop* or ongoing care or outpatient service* or peer support* or relapse prevention or relapse planning or social support* or support group* or through care or through-care wraparound or wrap-around or volunt*). |
| ((alternative* or community or mental health or holistic or humanistic or parenting skill* or prosocial or psychosocial or psychoeducation* or relapse prevention or relapse planning or resilienc* or restorative justice or self efficacy or social or strength* based or wellbeing or well being or vocational) adj3 (care or counsel* or intervention* or maintenance or practice* or program* or service* or support* or therap* or training or treat* or workshop*)). |

**Models of Care**

| (Delivery of care or model* of care or care model* or model pathway or service model or service provision or care pathway or provision of care or provision of service or good practice* or standard practice* or best practice*) |
| --- |
| (model* adj5 (care or delivery or pathway or provision or service*)) |

**Setting Terms**

| prisons/ or prisoners/ |
| --- |
| (jail* or prison* or incarcerat* or offender* or corrections or correctional) |

**Mental Health Terms**

| (mental disorder* or mental health or mental illness* or psycholog* or psychosocial or psychiatr*) |
| --- |
| mental health/ or psychology, applied/ or mental disorders/ or "behavioral disciplines and activities"/ or mental health services/ |

**Review (source) Terms**

| meta-analysis.pt. |
| --- |
| meta-analysis/ or systematic review/ or meta-analysis as topic/ or "meta analysis (topic)"/ or "systematic review (topic)"/ |
| ((systematic* adj3 (review* or overview*)) or (methodologic* adj3 (review* or overview*))) |
| ((quantitative adj3 (review* or overview* or synthes*)) or (research adj3 (integrati* or overview*))) |
| ((integrative adj3 (review* or overview*)) or (collaborative adj3 (review* or overview*)) or (pool* adj3 analy*)) |
| (data synthes* or data extraction* or data abstraction*) |
| (handsearch* or hand search*) |
| (standardize* or operationalize* or systematic) |
| (meta regression* or metaregression*) |
| (medline or cochrane or pubmed or medlars or embase or cinahl) |
| (cochrane or (health adj2 technology assessment) or evidence report) |
| (meta-analysis or systematic review) |
| (comparative adj3 (efficacy or effectiveness)) |
| (outcomes research or relative effectiveness) |
| ((indirect or indirect treatment or mixed-treatment) adj comparison*) |
| (synthesis or review or meta analy* or summary or overview) |

**Search Formulas**

**STA:** (STA search terms) AND (setting search terms) AND (mental health search terms) AND (review search terms)

**I:** (I search terms) AND (setting search terms) AND (mental health search terms) AND (review search terms)

**R:** (R search terms) AND (setting search terms) AND (mental health search terms) AND (review search terms)

**Models of Care:** (models of care search terms) AND (setting search terms) AND (mental health search terms)

**Validation articles retrieved (MedLine)**

**STA:**

1. Mental health screening tools in correctional institutions: a systematic review (Martin et al., 2013)
2. The evaluation of mental health screening practices within a population of incarcerated women (Gallagher et al., 2013).
3. Screening and access to services for individuals with serious mental illnesses in jails (Sheyett et al., 2009).

**I:**

1. Treating Offenders with Mental Illness: A Research Synthesis (Morgan et al., 2013)
2. Outcomes of Psychological Therapies for Prisoners With Mental Health Problems: A Systematic Review and Meta-Analysis (Yoon et al. 2017)
3. Providing treatment to prisoners with mental disorders: development of a policy: Selective literature review and expert consultation exercise (Earthrowl et al., 2018)

**R:**

1. Interventions at the Transition from Prison to the Community for Prisoners with Mental Illness: A Systematic Review (Hopkin et al., 2018)
2. Systematic review of qualitative evaluations of reentry programs addressing problematic drug use and mental health disorders amongst people transitioning from prison to communities (Kendall et al., 2018)
3. Mental Health Issues of Women After Release From Jail and Prison: A Systematic Review (Stanton et al., 2016)

**Models of Care:**

1. Mental illness and the provision of mental health services in prisons (Forrester et al., 2018)
2. From positive screen to engagement in treatment: a preliminary study of the impact of a new model of care for prisoners with serious mental illness (Pillai et al., 2016)
3. A model for the provision of jail mental health services: an integrative, community-based approach (Ogloff et al., 1991)
